# A Case of Chronic Elephantiasis Nostra Verrucosa Highlighting the Progression of Chronic Lymphedema

**DOI:** 10.7759/cureus.101352

**Published:** 2026-01-12

**Authors:** Sarah E Moffitt, Zachary M Schwartz, Himara Koelmeyer, Aditya Grover

**Affiliations:** 1 Internal Medicine, University of South Florida (USF) Health Morsani College of Medicine, Tampa, USA

**Keywords:** cellulitis, chronic lymphedema, elephantiasis nostra verrucosa, lichenification, morbid obesity

## Abstract

Elephantiasis nostra verrucosa (ENV) is a rare form of secondary, nonfilarial chronic lymphedema, typically linked to conditions such as morbid obesity, congestive heart failure, and chronic venous insufficiency (CVI). Bacterial infection is thought to initiate a vicious cycle of inflammation, limb swelling, and fibroblast proliferation that results in the “cobblestone” appearance of the affected extremity.

A 67-year-old male with a past medical history of end-stage renal disease (ESRD) on hemodialysis, morbid obesity, type 2 diabetes mellitus, and ENV presented to our ED with cellulitis of his right lower extremity after minimal trauma to his leg. The patient had a BMI of 60.7 kg/m², ENV of the bilateral lower extremities with cellulitis of the right leg, and annular psoriasis diffusely involving the bilateral arms, abdomen, back, head, and neck. Management of the right leg cellulitis included a CT scan to evaluate for osteomyelitis, IV vancomycin and cefepime for broad-spectrum coverage, and consultations with Nephrology and Dermatology to coordinate hemodialysis and antibiotic dosing in the setting of ESRD, and management of annular psoriasis, respectively. Patient education on hygiene for the lower extremities and fistula site was also provided. The patient was discharged after 3 days with outpatient Dermatology follow-up established.

ENV, although rare, presents a significant challenge to patients and physicians. Much of the literature on ENV derives from case reports, with limited data to elucidate what specifically provokes its development. This scarcity of literature creates challenges in incorporating evidence-based treatment regimens. As seen in our patient’s case, recommendations for ENV are typically conservative measures, such as weight loss, infection prevention, and lymphatic pumps or compression garments. The use of retinoids and surgical intervention has also been reported, with minimal improvement. Our case highlights the importance of preventing conditions such as ENV through early intervention and patient education.

## Introduction

Elephantiasis nostra verrucosa (ENV) is a rare form of secondary, nonfilarial chronic lymphedema, resulting from disruption of lymphatic drainage, typically in gravity-dependent body parts [1]. The term “nostra,” meaning “from our region,” was first used in 1933 by Sir Aldo Castellani in England to describe elephantiasis not of filarial etiology [2]. It is proposed that a variety of conditions can cause the development of ENV, including radiation, congestive heart failure (CHF), malignancy, trauma, obesity, hypothyroidism, and chronic venous stasis [1]. Although the pathophysiology of ENV is unclear, it is thought that bacterial infection plays a major role in igniting a vicious cycle of lymphatic stasis, fibroblast proliferation, limb swelling, and subsequent vulnerability to additional bacterial infections [3]. The resulting dermatologic consequence of chronic lymphedema, infection, and skin thickening is tissue with extensive hyperkeratosis, dermal fibrosis, lichenification, and a typical “cobblestone” appearance of the affected area [3].

The differential diagnosis for ENV includes venous stasis dermatitis, lipedema, lipodermatosclerosis, pretibial myxedema, and chromoblastomycosis [1]. Venous stasis dermatitis and lipedema can be quickly differentiated from ENV, as they do not exhibit the extensive hyperkeratosis that is associated with ENV [1]. Lipodermatosclerosis has a typical “inverted wine bottle” appearance attributed to chronic venous insufficiency (CVI) [1]. Hyperpigmentation is commonly seen in chronic phases [1]. Lipodermatosclerosis is more commonly seen in middle-aged women with venous insufficiency, contrasting with ENV, which is typically seen in morbidly obese patients [1]. Pretibial myxedema is nearly exclusively linked to Graves’ disease and lacks the verrucous hyperkeratosis of ENV [1]. Chromoblastomycosis may have similar physical examination features to ENV, but it is a fungal infection following traumatic inoculation [1]. Distinguishing this from ENV can be accomplished through patient history and histologic examination [1].

We present a case report of a patient with ENV secondary to morbid obesity to add to the literature.

## Case presentation

**Figure 1:**
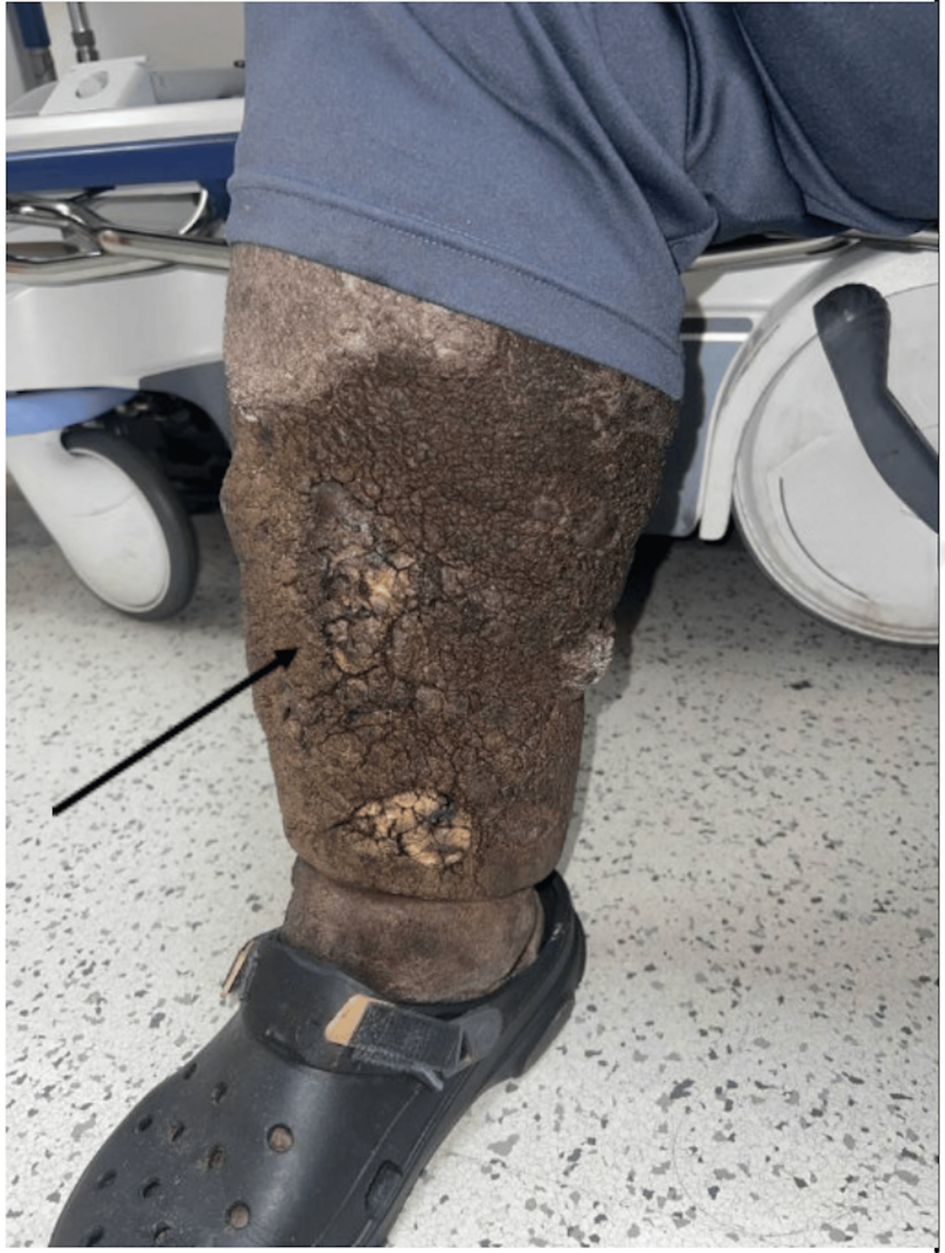
Stage 3 lymphedema of the right lower extremity with overlying cellulitis and chronic changes secondary to ENV. ENV: Elephantiasis nostra verrucosa.

A 67-year-old male with a past medical history of end-stage renal disease (ESRD) on hemodialysis, morbid obesity, type 2 diabetes mellitus, and ENV presented to our ED with cellulitis of his right lower extremity after minimal trauma to his leg. Upon physical examination, the patient was morbidly obese with a BMI of 60.7 kg/m², Stage 3 lymphedema of the lower extremities, and hypopigmentation of the skin on the face and neck. The lower extremities were circumferentially lichenified with papillomatosis and hypopigmented plaques scattered along the legs. The right lower extremity had an area of yellow, hardened discoloration corresponding to the location of the suspected cellulitis (Figure 1).

**Figure 2:**
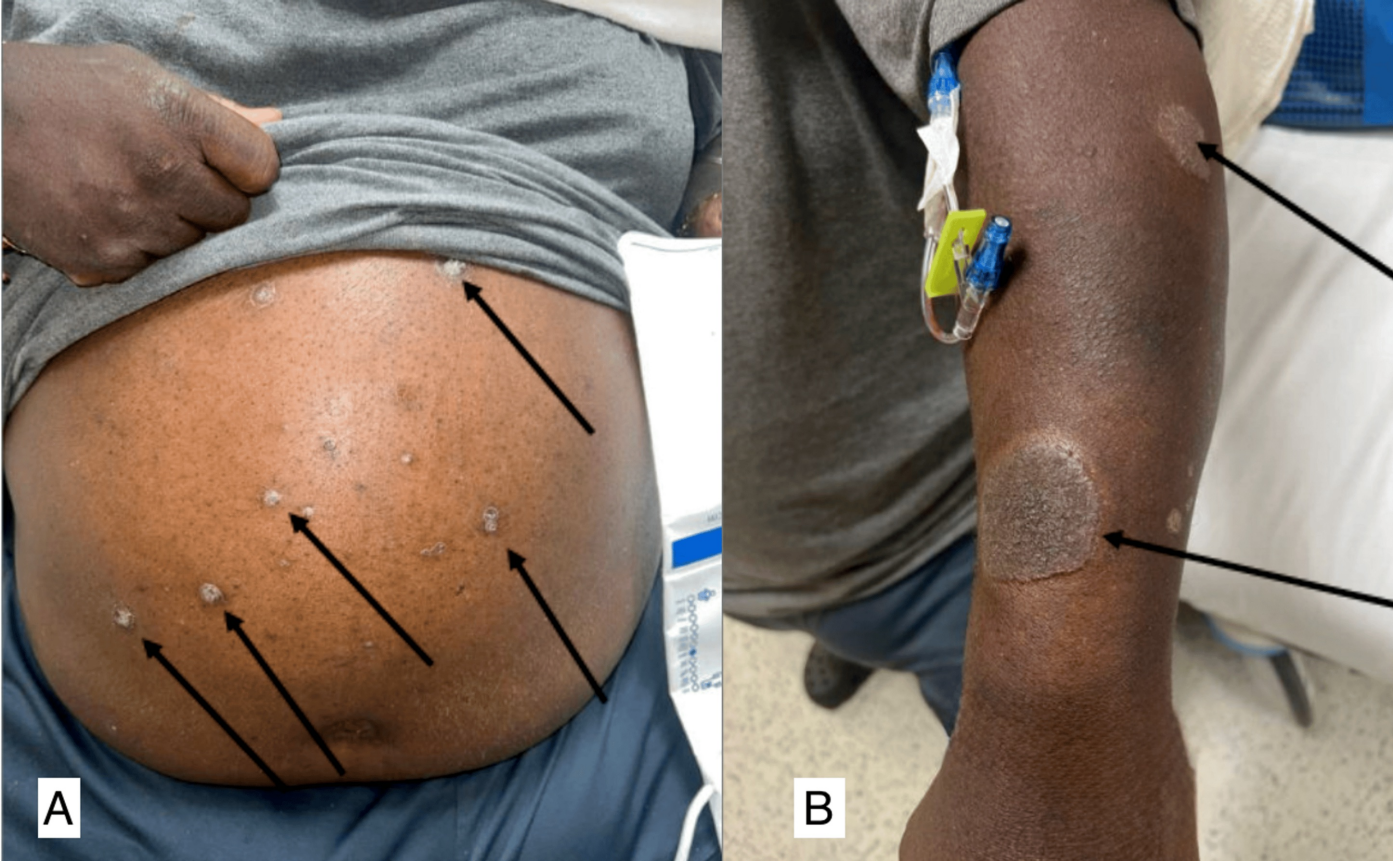
(A) Plaque psoriasis with a possible annular configuration on the abdomen. (B) Plaque psoriasis on the left arm (arrows indicate affected areas).

The patient also reported a mildly pruritic, annular rash of various sizes that sometimes bled on his arms and abdomen for the past four to six weeks (Figure 2).

He stated that this has been an annual issue for him, with his symptoms presenting consistently when the temperature increases significantly. Additionally, the patient reported that he had not been showering regularly for fear of damaging his hemodialysis line. Our initial differential diagnosis included overlying cellulitis on ENV, with other dermatologic conditions such as lipodermatosclerosis and pretibial myxedema. We felt these other diagnoses were less likely, as lipodermatosclerosis and pretibial myxedema are typical of different patient populations, such as middle-aged women and patients with Graves’ disease, respectively. Moreover, these conditions lack the highly characteristic hyperkeratosis of ENV.

**Figure 3:**
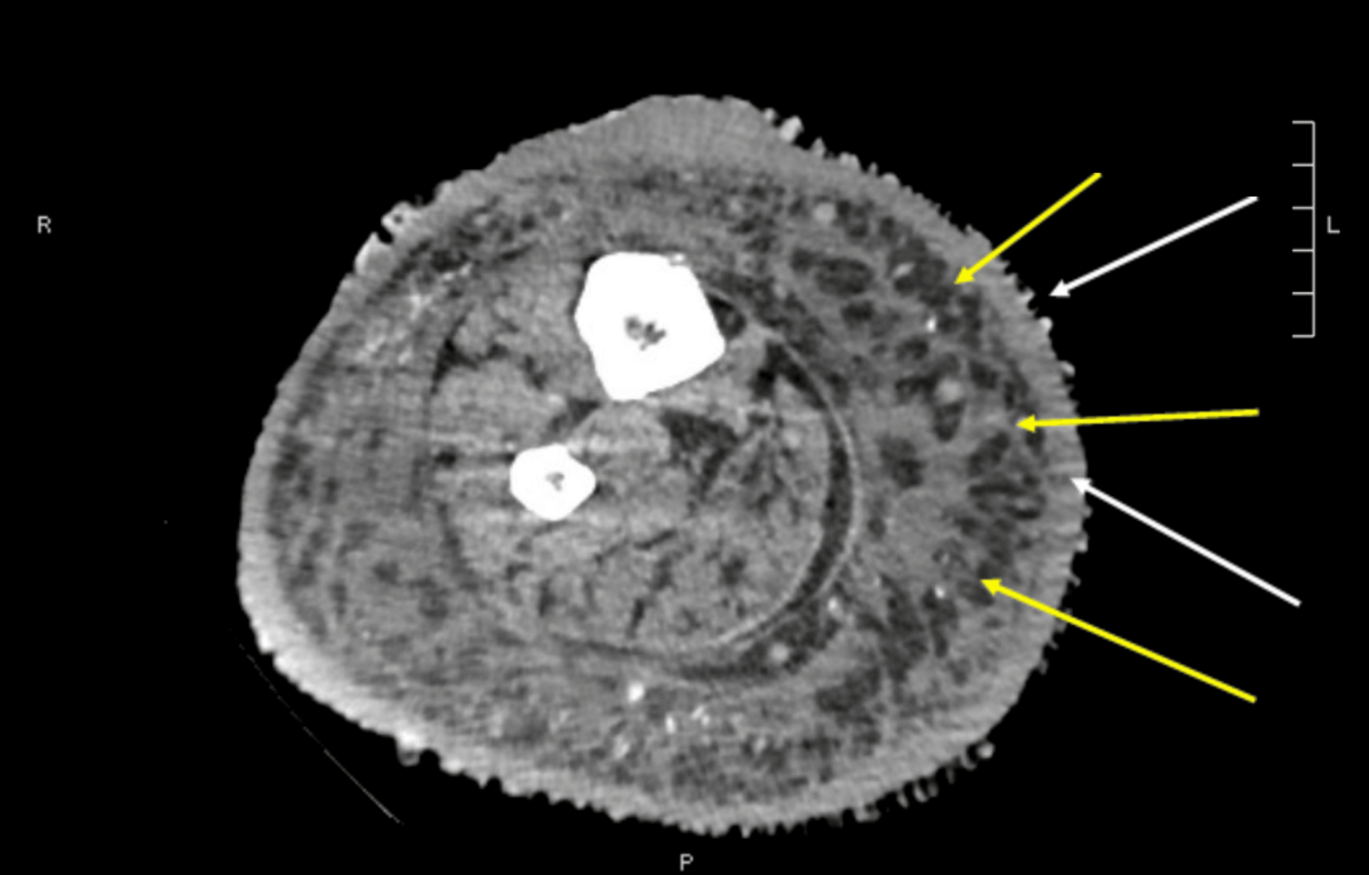
CT scan of the right lower extremity demonstrating hyperkeratosis (white arrows) and subcutaneous bullae (yellow arrows), characteristic features of ENV. ENV: Elephantiasis nostra verrucosa.

His admitting CBC and complete metabolic panel (CMP) were largely unremarkable other than a blood urea nitrogen (BUN) of 49 and a creatinine of 10.7. The patient reported that he had missed dialysis on the day of admission. A CT scan with contrast of the right lower extremity was ordered to further evaluate for osteomyelitis (Figure 3).

Nephrology was consulted for antibiotic dosing and timing recommendations in the setting of ESRD and hemodialysis. The patient was started on an antibiotic regimen including vancomycin and cefepime for broad-spectrum coverage. Dermatology was consulted for his diffuse skin rash, which covered ~35% of his body surface area. They prescribed topical ketoconazole with hydrocortisone and triamcinolone for his rash, which was determined to be psoriasis, and recommended conservative management (exercise, lymphatic massage, and compression devices) to help reduce his chronic venous stasis.

The CT scan was negative for osteomyelitis but noted a potential overlying soft tissue infection. Blood and wound cultures were not obtained. The patient’s condition remained stable during his hospitalization, and a plan was made to transition him to oral antibiotics after a three-day admission. The patient was started on per os trimethoprim-sulfamethoxazole (TMP-SMX), and follow-up with Dermatology was scheduled. Additionally, education on proper management of the patient’s lymphedema, chronic venous stasis, and hemodialysis line hygiene was provided. Case management was consulted to assist with the patient’s reported financial strain.

The patient continues to follow with Dermatology for ENV and psoriasis. Dermatology recommended compression bandages, lymphatic massage, and topical Selsun Blue daily for the hyperkeratosis. Additionally, Dermatology initiated the patient on risankizumab and topical antifungals for the psoriasis. They recommended another Bariatric Surgery consultation now that he has lost weight to 399 lb as of January 2025. A summary of the patient’s laboratory, imaging, and physical examination findings is reported in Table 1.

**Table 1:** Laboratory results and physical examination upon admission. BUN: Blood urea nitrogen.

Lab/Imaging/Physical Exam	Admission
Hemoglobin	9.8
WBC	7.2
BUN	49
Creatinine (Cr)	10.7
CT scan	Severe, diffuse subcutaneous soft-tissue edema in the right lower leg with skin thickening and superficial bullous collections, similar to February 5, 2024. Findings may reflect venous stasis or lymphedema, with or without overlying cellulitis. No abscess. No acute fracture or CT evidence of osteomyelitis. Periostitis of the tibia and fibula, similar to prior, likely reactive or secondary to venous stasis/lymphedema.
Physical exam findings	Well-demarcated erythematous to hyperpigmented scaly plaques on the face, scalp, and neck. Round hyperpigmented, somewhat velvety scaly plaques scattered on the upper arms and mid and lower back, sparing the buttocks and elbows. Dark brown lichenified papules on the abdomen. Massive edema and induration of the inferior pannus bilaterally. Marked edema, induration, and flesh-colored papillomatosis of the bilateral lower legs, with an area of likely infection noted in the right lower extremity (RLE) below the knee.

History of lymphedema

**Figure 4:**
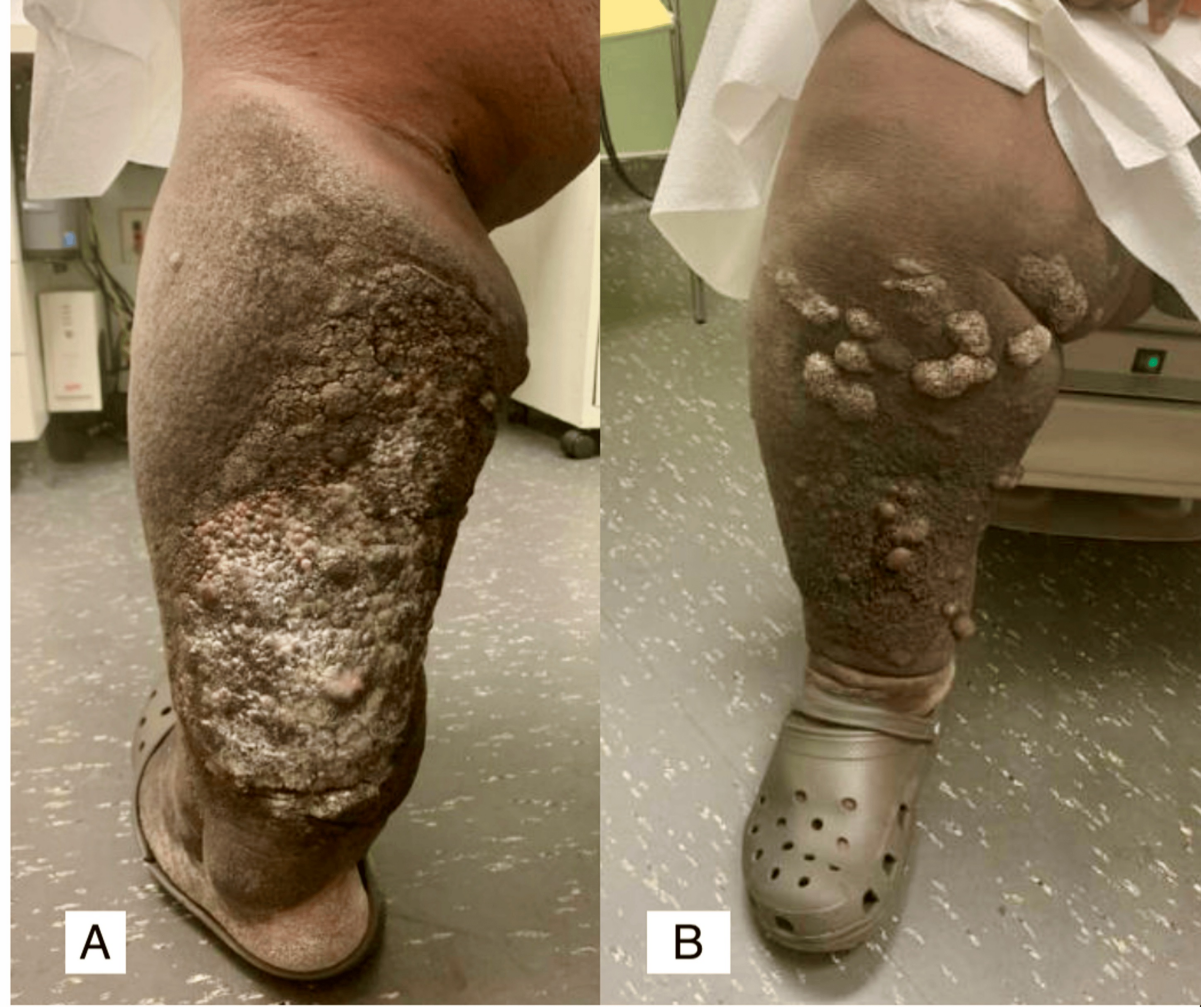
(A) Initial presentation to our hospital with significant bilateral lower extremity lymphedema and extensive chronic changes, with overlying cellulitis on the posterior left leg. (B) Anterior view of the right lower extremity with chronic lymphedema.

Further investigation into the patient’s past medical history revealed extensive chronic lymphedema of the bilateral lower extremities (BLE) for at least seven years. At that time, the patient was morbidly obese (BMI 65 kg/m²) with hypertension (HTN) and Stage 4 chronic kidney disease (CKD). His first presentation to our hospital for lower extremity lymphedema was in July 2021 (Figure 4).

**Figure 5:**
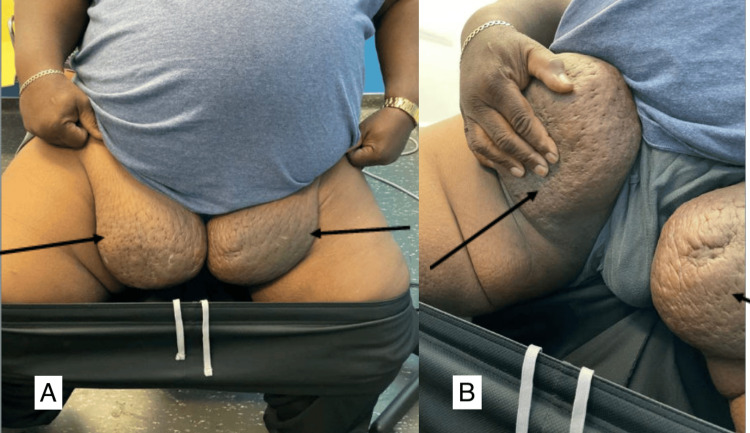
(A) Arrows indicate bilateral groin masses, likely secondary to chronic lymphedema, at the initial Plastic Surgery consultation in 2021. (B) Arrows show elevation of the right groin mass and the left groin mass.

He was evaluated by the Plastic Surgery team to discuss bilateral groin masses that had been enlarging over two years (Figure 5).

The etiology of these masses was presumed to be a consequence of chronic lymphedema, as the patient attempted to alleviate the swelling by wrapping his legs with All Cotton Elastic (ACE) bandages. The patient was referred to the Bariatric Surgery clinic to discuss weight loss options before operative intervention of his groin was possible. He followed with the Bariatric Surgery clinic through 2023. He also initiated manual lymphatic drainage therapy and home lymphatic pumps during this time, with no resolution. His overall health declined during this period, notably his kidney function. He progressed to CKD Stage 5 and began hemodialysis in June 2023. He underwent a cardiac evaluation for surgical clearance in October 2023. His cardiologic status was indeterminate and warranted further testing, as his routine EKG depicted lateral T-wave inversions. However, the patient was lost to follow-up with the Cardiology and Bariatric Surgery clinics after October 2023.

Ultimately, the patient never underwent bariatric surgery and did not see improvement in his BLE. Moreover, his bilateral groin masses were unable to be operated on due to his BMI. The patient therefore had recurrent bouts of soft tissue infection of his lower extremities, with multiple hospital admissions for imaging to rule out osteomyelitis and observation on antibiotics in the setting of CKD Stage 5 and hemodialysis.

**Figure 6:**
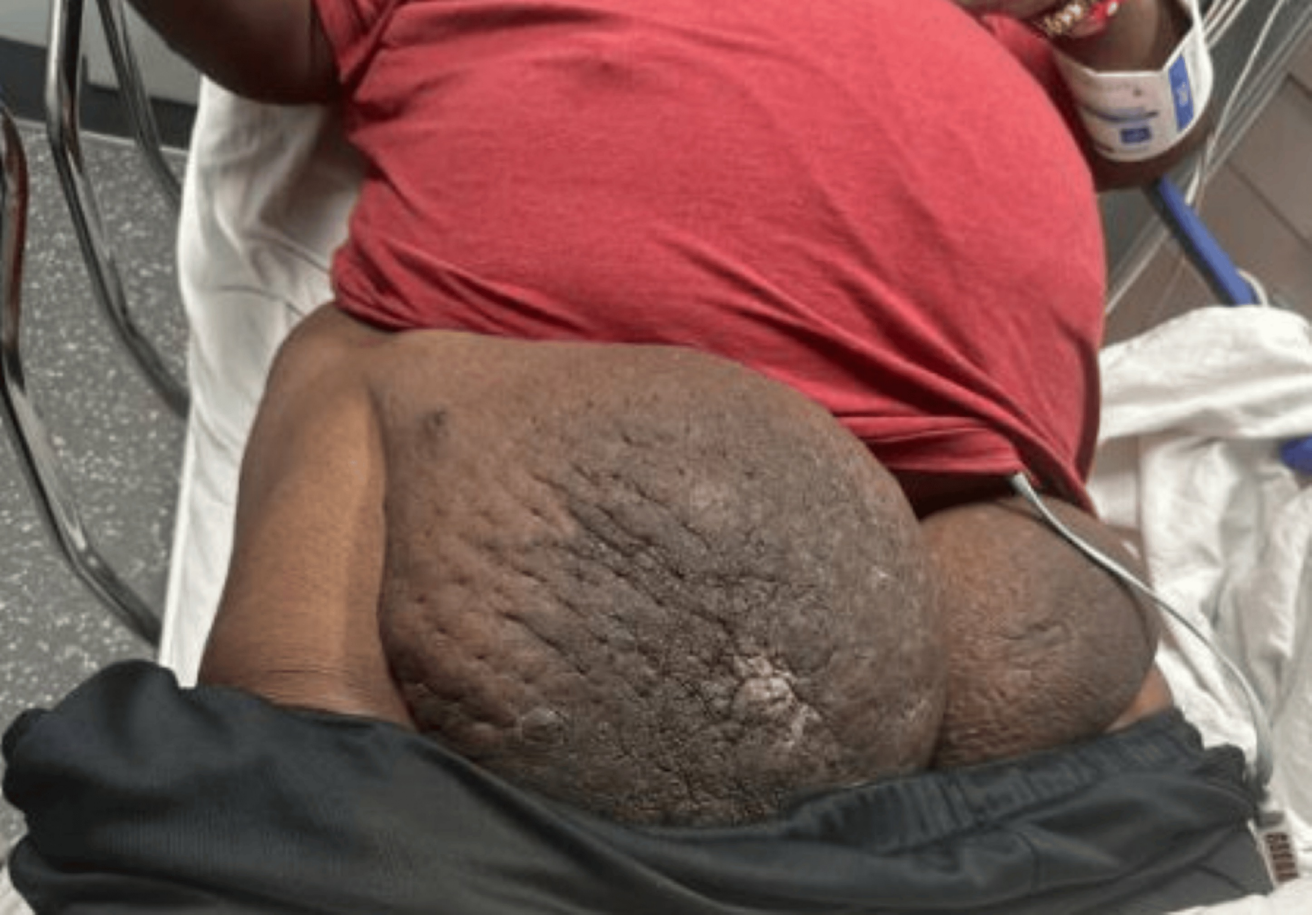
Bilateral groin masses at the February 2025 General Surgery evaluation. The right mass shows significant interval growth compared with the left mass and with the initial presentation four years earlier.

In February 2025, the patient returned to the hospital and was seen by General Surgery to evaluate the possibility of excising his right groin mass (Figure 6).

The right groin mass had grown considerably over the years and severely impeded his ability to ambulate. Soft tissue resection of the right groin mass was completed in conjunction with the Plastic Surgery team in April 2025. The right groin mass weighed 5,500 g and was sent for surgical pathology, along with a right inguinal lymph node. Both the groin mass and lymph node were benign but did demonstrate marked fibrosis consistent with chronic lymphedema. The patient healed well postoperatively and inquired about removal of the left groin mass. The patient was advised to continue losing weight, and the team will consider surgical excision when the patient is around 300 lb. As of October 2025, the patient remained at 388 lb.

## Discussion

ENV, although rare, presents a significant challenge to patients and physicians [3, 4]. Much of the literature on ENV derives from case reports, with limited data to elucidate what specifically provokes its development [3]. A retrospective chart review from 2006 to 2008 reported on 21 patients with ENV to analyze contributing factors [3]. In this study, all 21 patients were obese (mean BMI 55.8), with 91% in the range of morbid obesity [3]. CVI was present in 15/21 patients, and 18/21 patients reported a history of soft tissue infection of the affected areas and bilateral leg involvement [3]. ENV of the calves (81%) was more common than involvement of the thighs (19%) or abdomen (9.5%) [3]. Other case reports also discuss CHF as a contributing factor to ENV [1, 2, 4].

The scarcity of literature on this disease creates a challenge for incorporating evidence-based treatment regimens [3, 4]. As seen in our patient’s case, recommendations for ENV are typically weight loss, infection prevention, and lymphatic pumps or compression garments [3]. There have been case reports on the use of retinoids, notably acitretin, decreasing the size of skin lesions by decreasing epidermal proliferation and inflammation [4-7]. Surgical intervention, such as debridement of the affected skin or lymphovenous anastomosis bypass, has controversial outcomes in the literature [5]. Surgical treatments have not been shown in some cases to have long-term impact, likely due to delayed presentation and the chronic nature of ENV [5]. However, a case report from 2004 describing a technique to debride the verrucous lesions with a dermatome and dermabrasion successfully treated the cutaneous symptoms [8]. Amputation has even been recommended in cases with severe morbidity, such as osteomyelitis infection of the underlying bone [9].

Our case presents a strong example of the importance of preventative care and early intervention. Moreover, our patient’s financial strain greatly impacted his ability to seek and maintain care. Educating our patients and the overall community before they arrive in the hospital with chronic conditions such as CHF, CVI, and morbid obesity could help prevent the development of ENV. As illustrated by our patient’s case and previous case reports, ENV is extremely difficult to treat and rarely shows noticeable improvement with conservative or more invasive management. Although a rare manifestation of chronic morbid obesity and lymphatic dysregulation, the consequences of ENV have a serious impact on a patient’s quality of life. Our patient discussed surgery multiple times with our medical team during his admission and sought out surgical options for his large bilateral groin masses that resulted from chronic lymphedema. The combination of his BMI, hemodialysis, low exercise tolerance, and obstructing bilateral groin masses created an extremely limiting situation for our patient to lose enough weight to be considered for elective surgery. Additionally, his delayed presentation to the Bariatric Surgery clinic lessened his chances of being medically optimized for the operation. However, after years of struggling with the bilateral groin masses, he ultimately underwent resection of his right groin mass with the General Surgery and Plastic Surgery teams in 2025. Careful consideration and coordination with Cardiology, Nephrology, and Anesthesia were required to ensure a safe operation given the patient’s comorbidities.

## Conclusions

ENV is uncommon but may be preventable in some cases if inflammation, venous insufficiency, and infections can be effectively managed. Case reports in the literature commonly describe patient factors that promote chronic inflammation, including obesity, CVI, CHF, and soft tissue infection. This patient’s case includes all of these factors, and builds on them, as his follow-up over many years with various specialists demonstrates how difficult ENV is to treat. Additionally, this patient’s unique case aspects (atypical groin involvement, ESRD on dialysis) add further potential complications of ENV, as well as treatment considerations, to the existing literature. As an effective treatment for this condition has yet to be found, the best strategy is prevention through consistent patient and community education regarding obesity, lymphedema, hygiene, and cardiovascular health.
